# Realizing Tao-Thouless-like state in fractional quantum spin Hall effect

**DOI:** 10.1038/srep33472

**Published:** 2016-09-21

**Authors:** Chen-Rong Liu, Yao-Wu Guo, Zhuo-Jun Li, Wei Li, Yan Chen

**Affiliations:** 1 Department of Physics and State Key Laboratory of Surface Physics, Fudan University, Shanghai 200433, China; 2 State Key Laboratory of Functional Materials for Informatics and Shanghai Center for Superconductivity, Shanghai Institute of Microsystem and Information Technology, Chinese Academy of Sciences, Shanghai 200050, China; 3 CAS Center for Excellence in Superconducting Electronics, Shanghai 200050, China; 4 Collaborative Innovation Center of Advanced Microstructures, Nanjing 210093, China

## Abstract

The quest for exotic quantum states of matter has become one of the most challenging tasks in modern condensed matter communications. Interplay between topology and strong electron-electron interactions leads to lots of fascinating effects since the discovery of the fractional quantum Hall effect. Here, we theoretically study the Rashba-type spin-orbit coupling effect on a fractional quantum spin Hall system by means of finite size exact diagonalization. Numerical evidences from the ground degeneracies, states evolutions, entanglement spectra, and static structure factor calculations demonstrate that non-trivial fractional topological Tao-Thouless-like quantum state can be realized in the fractional quantum spin Hall effect in a thin torus geometric structure by tuning the strength of spin-orbit coupling. Furthermore, the experimental realization of the Tao-Thouless-like state as well as its evolution in optical lattices are also proposed. The importance of this prediction provides significant insight into the realization of exotic topological quantum states in optical lattice, and also opens a route for exploring the exotic quantum states in condensed matters in future.

Since the discovery of spin-Hall effect in 2004^1^,^2^, the study of spin-orbit coupling effects on quantum physics has been triggered great research interests both in condensed matter community and material science in this decade, especially fueled by the realization of the time reversal invariant topological insulators^3^,^4^ and Weyl semimetals^5^,^6^, leading to the most challenging task of the search for exotic quantum states and their realizations in modern condensed matter physics. Learned from the physics of fractional quantum Hall effects^7^, interplay between topology and strong electron-electron interactions displays lots of fascinating effects. The prominent fractional quantum Hall states occurring at certain unique values of the filling factor 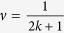, *k* integer, have been explained by Laughlin as an “incompressible quantum fluid state”^8^. Subsequently, Tao and Thouless^9^ proposed an alternative ground state with a gap to excitations, which has a charge-density-wave (CDW)-like structure and can be connected to the Laughlin’s state by an adiabatic change of the aspect ratio 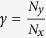
10,11,12,13,14 without undergoing a quantum phase transition. Namely, Tao-Thouless state is the exact ground state once *N*_*x*_ → *O*(1). In addition, the stripe formation of conventional CDW state in a fractional quantum Hall system has also been argued for systems with even denominator filling factors^15^,^16^,^17^,^18^. It is crucial to point out that the main difference between conventional CDW and Tao-Thouless states is that the latter state associates non-vanished fractional quantum Hall conductance, while the former one does not. Importantly, these studies show the richness of quasiparticle phases in the fractional quantum Hall systems.

In recent years, the fractional quantum (spin) Hall effects^19^,^20^ have been realized theoretically in the fractional Chern insulators^21^,^22^,^23^,^24^ without an external magnetic field, where strongly interacting particles partially fill up the topological flat-band with nonzero Chern number^21^,^25^,^26^. The stability of the edge states in fractional quantum spin Hall systems against interactions and disorders was analyzed by Levin and Stern^20^: A criterion *σ*_*sH*_/e* (*σ*_*sH*_ the spin Hall conductance and e* the Abelian quasi-particles of charge), is proposed to determine whether the system is a non-trivial fractional topological insulator or not. Neupert *et al*.^27^ studied the stability of fermionic fractional topological insulating phase with the filling factor 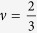 and pointed out that the system favors a fractional quantum spin Hall state for two decoupled spin species, but it should lead to an unstable fractional topological insulating phase according to Levin and Stern’s criterion. In fact, the fractional quantum spin Hall effect can possess fractionalized excitations in the bulk irrespective of the existence of gapless edge modes^28^. Moreover, by tuning the inter-spin interaction, the fractional quantum spin Hall state evolves into a conventional CDW stripe phase^27^,^28^,^29^,^30^ through a phase transition signalled by the closing of energy and quasi-spin excitation gaps^30^. Furthermore, Bosonic analogues of those fractional topological insulators have also been extensively studied by Repellin *et al*.^31^, and were found to be robust to perturbations in the bulk by introducing a spin-orbit coupling.

In this Letter, we propose a theoretical realization of fractionalized topological Tao-Thouless-like quantum state in a fractional quantum spin Hall system with a thin torus geometric structure by tuning the strength of Rashba-type spin-orbit coupling based on the framework of finite size exact diagonalization method. The obtained Tao-Thouless-like state has the property of time reversal symmetry, which is a counterpart of Tao-Thouless state in fractional quantum Hall systems^9^ or in fractional Chern insulators^32^ with time reversal symmetry breaking. Additionally, we also present a discussion of the possible experimental realization and detection of the Tao-Thouless-like topological quantum state as well as its evolution in optical lattices.

## Results

### The dispersion of single-particle bands

**Figure 1:**
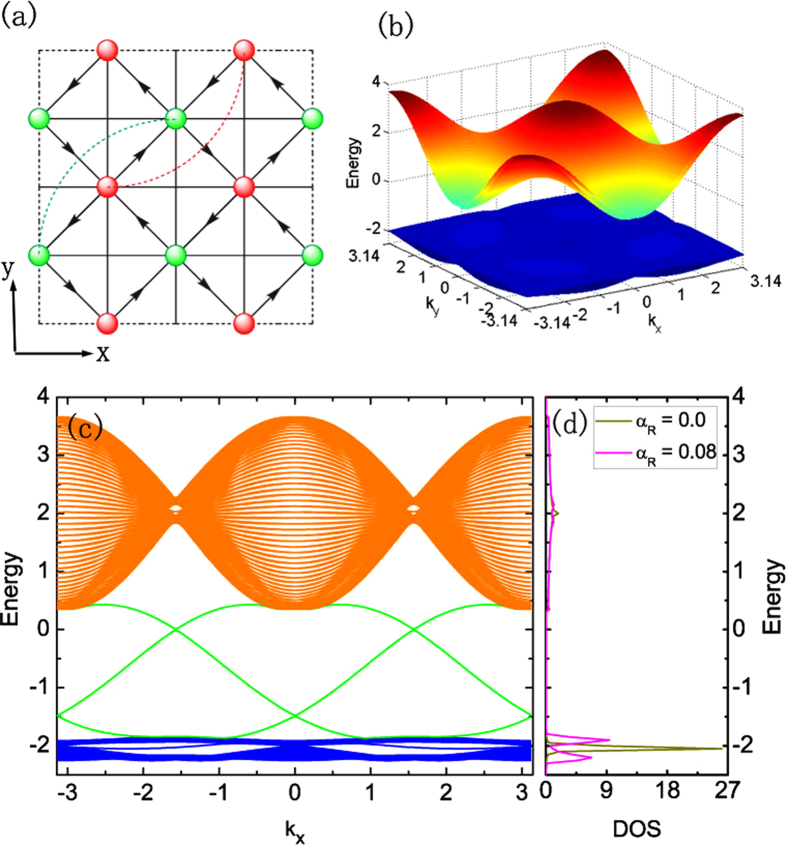
Lattice structure and electronic behaviours of single-particle Hamiltonian. (**a**) The checkerboard lattice structure of the flat-bands model, with arrows and (solid and dashed) lines representing the NN and NNN hoppings, respectively. The direction of the arrow shows the sign of the phase in the NN hopping terms. Two of the NNN hoppings are shown as the dashed curve. By putting the system on a torus and a cylinder, the single-particle energy dispersion with the strength of Rashba spin-orbit coupling *α*_*R*_ = 0.08 are shown in (**b**,**c**), respectively. In (**c**), the helical edge states (green lines) protected by time reversal symmetry are observed. (**d**) The density of states (DOS) on a torus structure with Rashba spin-orbit coupling *α*_*R*_ = 0.0 and *α*_*R*_ = 0.08.

The single-particle band dispersion of the Hamiltonian 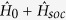 (see model Hamiltonian in Methods) on the system with torus and cylinder structures are shown in Fig. 1(b,c), which have a large bulk energy gap with the amplitude of 2*t*_1_ well separating the two spin-mixed flat-bands and conduction bands. Introducing the spin-orbit coupling the system still keeps the time reversal invariance but the inversion symmetry is broken, the two spin degenerate flat-bands will be split [see Fig. 1(d)] except at the time reversal invariant points. It is interesting to point out that there are some helical edge states emerging inside the bulk energy gap and crossing each other at the Γ(*k*_*x*_ = 0) point forming the Dirac-like dispersion relation protected by time reversal symmetry, similar to the band structure of a topological band insulator^33^. As the bulk energy gap is much larger than the energy scale of the interactions, we can safely project Hamiltonian 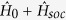 onto the states in the lowest two spin-mixed flat-bands in the exact diagonalization using a torus geometric structure. The repulsive interaction defined in Hamiltonian (1) (see model Hamiltonian in Methods) include a NN term which is parameterized by the coupling *V* and the dimensionless number *λ*. Previous studies^27^,^28^,^29^,^30^ have pointed out that the system favors a fractional quantum spin Hall and a conventional CDW stripe phases at small and large values *λ* of interspin interaction.

### The ground state properties of many-body Hamiltonian

**Figure 2:**
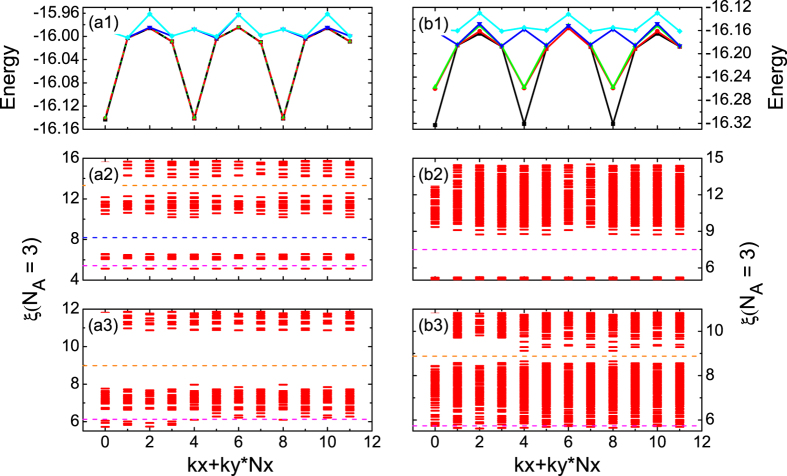
Ground state degeneracies and entanglement spectra of many-body Hamiltonian. (Top row) Ground state degeneracies for a system size *N*_*s*_ = 2 × *N*_*x*_(=2) × *N*_*y*_(=6), and the PES probing the *N*_*A*_ = 3 quasihole excitations for the *N*_*e*_ = 8 particles for a system size, (second row) *N*_*s*_ = 2 × *N*_*x*_(=2) × *N*_*y*_(=6), and (bottom row) *N*_*s*_ = 2 × *N*_*x*_(=4) × *N*_*y*_(=3). The Rashba-type spin-orbit coupling parameter *α*_*R*_ = 0 for ninefold state on the left column (**a**), and *α*_*R*_ = 0.08 for threefold state on the right column (**b**). In (**a2**,**a3**,**b3**), the states below the PES gap match the fractional quantum spin Hall state counting rule, while the one in (**b2**) matches the counting rule for a conventional CDW state.

The ground state spectra of the effect of the Rashba spin-orbit coupling *α*_*R*_ (see model Hamiltonian in Methods) on a fractional quantum spin Hall state are displayed in the top row of Fig. 2, where the parameters are chosen as *α*_*R*_ = 0 and *α*_*R*_ = 0.08 for (a1) and (b1), respectively, and shown that the ground state manifold is defined as a set of lowest states [nine-fold degeneracies in (a1) and three-fold degeneracies in (b1)] well separated from other excited states by a clear energy gap. Here it should be pointed out that the results [Fig. 2(a1] have been reported in our previous studies (see refs 29 and 30), we still present here to facilitate the following discussion concerned to the state evolution by applying the effect of spin-orbit coupling on the fractional quantum spin Hall state. From Fig. 2(a1, we also notice that the energy gap is always significantly larger than the energy splitting of the ground states for various system sizes. Although these states are not exactly degenerate on a finite system, their energy difference should fall off exponentially as the system size increases. In addition, it is interesting to find that for those states with three-fold or nine-fold degeneracy, if (*k*_*x*_, *k*_*y*_) is the momentum sector for one of the states in the ground states manifold, then the other state should be obtained in the sector (*k*_*x*_ + *N*_*e*_, *k*_*y*_ + *N*_*e*_) [modulo (*N*_*x*_, *N*_*y*_)], similar to that in fractional Chern insulators^22^. The relationship of the quantum numbers of the ground states manifold steams from the topological nontrivial characteristics^27^,^30^, which can be confirmed by the calculations of spectral flow [see the supplementary information (SI) for details].

### Entanglement spectra of ground states

We turn to reveal the nature of these states shown in the top row of Fig. 2 by using a powerful tool of particle entanglement spectra (PES)^23^,^34^,^35^,^36^,^37^, which provides an independent signature of the excitation structure of system as a fingerprint and remaining their characteristics in the thermodynamic limit. The entanglement energy levels *ξ* can then be displayed in groups labeled by the total momentum (*k*_*x*_, *k*_*y*_) for the *N*_*A*_ particles, and shown in Fig. 2(a2. When the entanglement spectrum is gapped, the number of states below the gap is a signature of a given topological phase, which is tightly related to the number of quasi-hole excitations, a hallmark of the fractional phase^23^,^31^,^38^. In our previous study^29^, we have demonstrated the nature of fractional quantum spin Hall state in Fig. 2(a2), which follows the counting rule of fractional quantum spin Hall state^30^,^31^. However, in Fig. 2(b2), the number of states below the gap of PES equalling to 168 deviates from the counting rule of fractional quantum spin Hall state, but precisely matches the conventional CDW counting^32^,^39^: 
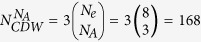
. Therefore, this result suggests that such state is a conventional CDW or CDW-like state.

To further well-understood the behaviors of the state shown in Fig. 2(b1), we adopt the aspect ratio dependent calculation of the PES. By tuning the aspect ratio 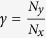 in a fixed system size from a thin torus 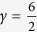 to a more two dimensional one 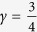, the calculated PES are shown in Fig. 2(a3. As a reference, we compare with Fig. 2(a2, and notice that the PES for the fractional quantum spin Hall state in both cases clearly display the similar gapped structure as well as sharing the same counting numbers below the gap of PES, as expected from our intuition. Moreover, comparing with Fig. 2(b2 as well as (a3), it is surprised that since changing the aspect ratio to a more two dimensional torus 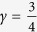 the structure of PES in Fig. 2(b3) is similar to that in Fig. 2(a3) rather than that in Fig. 2(b2), and the states below the gap of PES in Fig. 2(b3) is no long matching the conventional CDW counting instead by the one for fractional quantum spin Hall state. Therefore, it suggests that such CDW-like phase obtained in a thin torus geometric structure is indeed the Tao-Thouless-like state signalled by connecting to fractional quantum spin Hall state through an adiabatic change of the aspect ratio [see Fig. 2(b2] and evolved from fractional quantum spin Hall state without undergoing a quantum phase transition (see SI for details).

### Static structure factor

**Figure 3:**
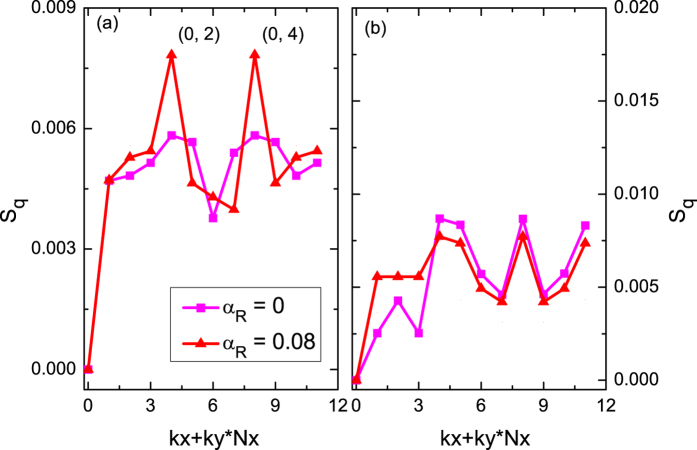
Static structure factor of ground state of many-body Hamiltonian. The SSF calculations *S*_**q**_ with various Rashba-type spin-orbit coupling parameter *α*_*R*_ for a system size (**a**) *N*_*s*_ = 2 × *N*_*x*_(=2) × *N*_*y*_(=6) and (**b**) *N*_*s*_ = 2 × *N*_*x*_(=4) × *N*_*y*_(=3).

In addition, we also present the static structure factor (SSF) calculations to further solidify our findings, and shown in Fig. 3(a). It is clearly shown that the Tao-Thouless-like state has a typical feature of CDW stripe order displaying the double unidirectional Bragg peaks aligned along the *x*-direction appeared at momenta **q** = **Q**_**1**_ [=(0, 2)] and **Q**_**2**_[=(0, 4)] in the SSF *S*_**q**_ calculation with the Rashba-type spin-orbit coupling strength *α*_*R*_ = 0.08 in the thin torus geometric structure with 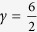. Furthermore, by tuning the aspect ratio *γ* to a more two dimensional torus geometric structure 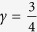, shown in Fig. 3(b), the double unidirectional peaks in SSF *S*_**q**_ calculation of the Tao-Thouless-like state is disappeared and instead by a featureless characteristic. By comparison with the featureless in SSF *S*_**q**_ calculation of the fractional quantum spin Hall state^29^, it suggests the phase, which is sensitive to the shape of lattice geometric structure, is indeed a Tao-Thouless-like state. All these obtained results are consistent with expected from the PES calculations and further solidify our findings.

## Experimental realizations

We propose an experimental realization of state of Tao-Thouless-like in future. Interestingly, it might be to study the spin-orbit coupling effect on the fractional quantum spin Hall state and obtain the state evolution. Very recently, a scheme of direct experimental realization of Rashba-type spin-orbit coupling^40^ and topological Haldane model^41^ in optical lattices was proposed, which might be helpful to establish the spin-orbit coupling effects on the flat-bands model with time reversal symmetry or equivalent bilayer flat-bands model in experiments^42^. Considering the interaction strength can be easily tuned in cold atom setups, our work will provide guidance for the experimental realization of the Tao-Thouless-like state and its evolution as well as exciting many-body fractional topological phases.

## Methods

### Model Hamiltonian

We start with a theoretical model Hamiltonian of electrons on a checkerboard lattice^29^,^30^ shown in Fig. 1(a):
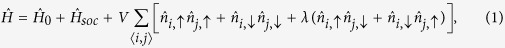
where 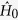 consists of two copies of *π*-flux phase with flat-bands as in ref. 26, and 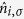 is the density operator on the site *i* with spin *σ*(=↑, ↓). In the single-particle part Hamiltonian 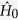, we denote 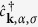 as the creation operator for an electron with lattice momentum **k** and spin *σ* in the sublattice *α* = *A*, *B*, and we introduce a spinor 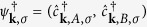. Then, the second quantized single-particle Hamiltonian 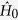 reads
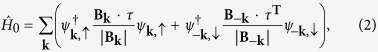
where the three vectors **B**_**k**_ are respectively defined as
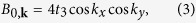

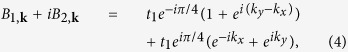

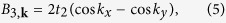
the identity and the triplet Pauli matrices *τ* = (*τ*_0_, *τ*_1_, *τ*_2_, *τ*_3_) act on the sublattice index. The parameters *t*_1_, *t*_2_, and *t*_3_ represent the nearest neighbor (NN) hopping, next-nearest-neighboring (NNN) hopping, and next-next nearest neighbor (third-NN) hopping amplitudes, respectively. In addition, the second term in Hamiltonian (1) describes the Rashba-type spin-orbit coupling and has the form^43^,^44^:
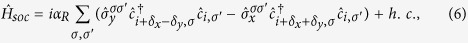
where *α*_*R*_ represents the strength of the Rashba-type spin-orbit coupling, *δ*_*x*_ and *δ*_*y*_ are the unit vectors along the 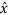 and 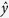 directions shown in Fig. 1(a).

### Many-body exact diagonalization

We exactly diagonalize the many-body Hamiltonian (1) projected to the lowest two flat-bands for a finite system with *N*_*x*_ × *N*_*y*_ unit cell (total number of sites *N*_*s*_ = 2 × *N*_*x*_ × *N*_*y*_) shown in Fig. 1(a). We denote the number of fermions as *N*_*e*_, and filling factor as 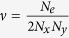. Because of the periodic boundary condition implementing translational symmetries, we diagonalize the system Hamiltonian in each total momentum 
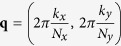
 sector with (*k*_*x*_, *k*_*y*_) as integer quantum numbers. Without loss of generality, we set the *t*_1_ as an energy unit and the interaction *V* = 1, *λ* = 0, and the filling factor 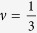 throughout this paper. Similar results for 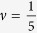-filling states can also be easily obtained when the NNN repulsion is included (see the SI for details).

### Entanglement spectra

We partition the system in the way as described in ref. 23 and divide the *N*_*e*_ particles into two subsystems of *N*_*A*_ and *N*_*B*_ particles, and trace out the degrees of freedom carried by the *N*_*B*_ particles. The eigenvalues *e*^−*ξ*^ of the resulting reduced density matrix *ρ*_*A*_ = *Tr*_*B*_*ρ*, where 
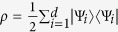
 is defined in a *d*-fold [nine-fold in Fig. 2(a1) and three-fold in Fig. 2(b1)] degenerate state |Ψ_*i*_〉.

### Static structure factor

The static structure factor is defined^30^,^45^,^46^,^47^ as
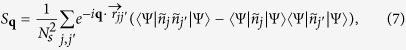
the 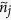 indicates the density at site *j* projected onto the lowest two flat-bands of single-particle Hamiltonian, and the wave function |Ψ〉 is incoherent summation over the degenerate ground states.

## Additional Information

**How to cite this article**: Liu, C.-R. *et al*. Realizing Tao-Thouless-like state in fractional quantum spin Hall effect. *Sci. Rep.* **6**, 33472; doi: 10.1038/srep33472 (2016).

## Supplementary Material

Supplementary Information
