# Supplementary material for: Realizing Tao-Thouless-like state in fractional quantum spin Hall effect
Source: Sci Rep. 2016 Sep 21;6:33472. doi: 10.1038/srep33472 (PMC5030710; doi:10.1038/srep33472)
Supplement: Supplementary Information [file srep33472-s1.pdf]

Supplementary Information of "Realizing Tao-Thouless-like state in fractional quantum spin Hall effect"

Chen-Rong Liu,[1] Yao-Wu Guo,[1] Zhuo-Jun Li,[2, 3] Wei Li,[2, 3, *] and Yan Chen[1, 4, †]

[1]*Department of Physics and State Key Laboratory of Surface Physics, Fudan University, Shanghai 200433, China*
[2]*State Key Laboratory of Functional Materials for Informatics and Shanghai Center for Superconductivity, Shanghai Institute of Microsystem and Information Technology, Chinese Academy of Sciences, Shanghai 200050, China*
[3]*CAS Center for Excellence in Superconducting Electronics, Shanghai 200050, China*
[4]*Collaborative Innovation Center of Advanced Microstructures, Nanjing 210093, China*

I. $\nu = \frac{1}{5}$ FILLING STATES

In order to study the $\nu = \frac{1}{5}$ filling states, one should further include the NNN repulsion interaction [1] into the Hamiltonian (1) in the main text, where the NNN repulsion interaction describes:

$$\hat{H}' = V' \sum_{\langle\langle i,j \rangle\rangle} [\hat{n}_{i,\uparrow}\hat{n}_{j,\uparrow} + \hat{n}_{i,\downarrow}\hat{n}_{j,\downarrow} + \lambda'(\hat{n}_{i,\uparrow}\hat{n}_{j,\downarrow} + \hat{n}_{i,\downarrow}\hat{n}_{j,\uparrow})].$$

Diagonalize the many-body Hamiltonian, we obtain the low energy spectra shown in Figs. 1(a1) and (b1), where the ground states manifold for the $\frac{1}{5}$-filling case are well defined as a set of lowest states [25fold degeneracies in (a1) and 5fold degeneracies in (b1)] well separated from other excited states by a clear energy gap. In addition, we also calculate the PES for corresponding ground state degeneracy and show in Figs. 1(a2) and (b2). Fig. 1(a2) clearly displays a fractional quantum spin Hall state with the number of states below the gap matched the fractional quantum spin Hall state counting [2], while the number of states below the PES gap in Fig. 1(b2) is 100, precisely matched with expected from the counting rule for conventional CDW state: [3, 4]: $N^{N_A}_{CDW} = 5 \begin{pmatrix} N_e \\ N_A \end{pmatrix} = 5 \begin{pmatrix} 6 \\ 3 \end{pmatrix} = 100$, similar

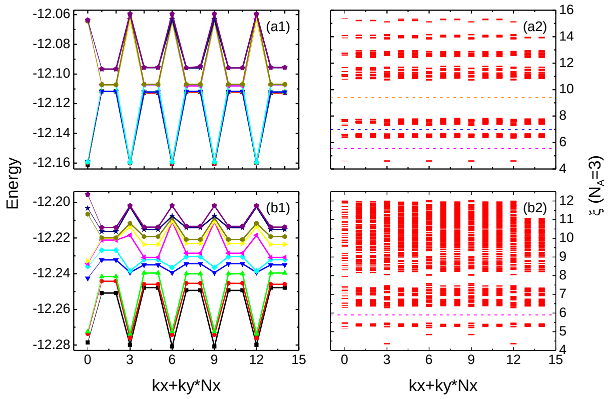

FIG. 1: (Color online) (a1) and (b1) Ground state degeneracies. (a2) and (b2) PES probing the $N_A = 3$ quasihole excitations for the $N_e = 6$ particles. The interaction $\alpha_R = 0$ for 25fold state on the first row, and $\alpha_R = 0.08$ for 5fold state on the bottom row. The system size is denoted as $N_s = 2 \times N_x(= 3) \times N_y(= 5)$, and the rest of interaction parameters are chosen as $U = 0$, $V = V' = 1$, and $\lambda' = 0$. In (a2) the states below the PES gap match the fractional quantum spin Hall state counting, while the one in (b2) matches the conventional CDW state counting.

*Electronic address: `liwei@mail.sim.ac.cn`
†Electronic address: `yanchen99@fudan.edu.cn`

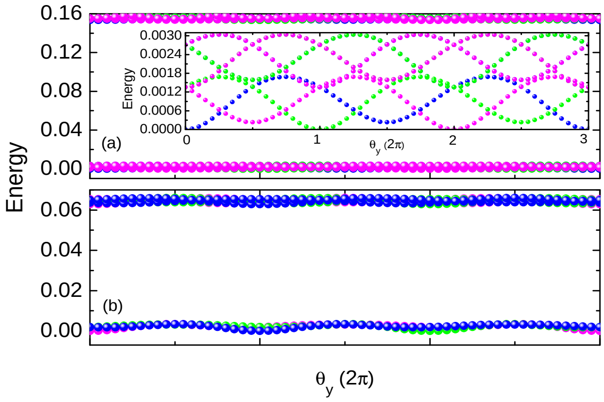

FIG. 2: (Color online) Evolution of low-lying energy spectra for (a) ninefold state with interaction $\alpha_R = 0$, and for (b) threefold state with $\alpha_R = 0.08$ upon flux insertion along the y-direction at $N_s = 2 \times N_x(= 2) \times N_y(= 6)$ lattices. Additionally, these ground state energies are all shifted by E_1, which is the lowest energy for the system.

to that obtained in $\nu = \frac{1}{3}$ filling case in the main text. Additionally, it should be noted that the state in Fig. 1(b1) is indeed a Tao-Thoules-like state, which can be connected to the fractional quantum spin Hall state through an adiabatic change of the aspect ratio without occurring a quantum phase transition.

II. SPECTRAL FLOW

To reveal the topological nontrivial state, we calculate the evolution of low-lying energy spectra by inserting a magnetic flux quantum in the system. For a many-body state [5, 6]: $|\Psi(\mathbf{r}_j)\rangle$, the twisted boundary condition in the y-direction is $|\Psi(\mathbf{r}_j + N_y\mathbf{a}_y)\rangle = e^{i\theta_y}|\Psi(\mathbf{r}_j)\rangle$, where θ_y is the boundary phase along y-direction and $\mathbf{a}_y$ is the lattice vector along the y-direction. According to Laughlin's gauge argument [7, 8]: For a $\nu(= 1/3)$ filling fractional quantum Hall system, when the flux adiabatically inserts $\frac{1}{\nu}(= 3)$ quantum fluxes, the states should evolve back to themselves looking exactly the same as before. From Fig. 2, we notice that the charge evolution spectra for both threefold and ninefold degenerate states share the same spectral flow pattern: both the three states and nine states are found to evolve into each other with level crossing and separated from the other low-energy excitation spectrum when imposing the boundary phases. Eventually, all levels return to their initial configuration after the insertion of three flux quanta. Previously we have obtained the spin Chern number $C_{sc} = \frac{2}{3}$ for states [9] in Fig. 2(a) by the many-body calculations [5, 10]. Due to the presence of spin-orbit coupling, the spin Chern number is no long well-defined in Fig. 2(b). Fortunately, this threefold state in Fig. 2(b) is indeed a Tao-Thouless-like state, which can be adiabatically connected to the fractional quantum spin Hall state without occurs a quantum phase transition by tuning the aspect ratio of geometric structure (see Fig. 3). Thus, the state evolution evidences that all those states still share the same topological index albeit the spin S_z is whether a good quantum number.

III. STATE EVOLUTIONS

We calculate the spectra of ground state to visualize the state evolutions from the fractional quantum spin Hall state to the Tao-Thouless-like state by tuning the Rashba spin-orbit coupling α_R, and shown in Fig. 3. It is demonstrated that the fractional quantum spin Hall state (9fold degenerate state in $\nu = \frac{1}{3}$ filling case) will be separated into two groups, one has much higher six-fold degenerate state in energy and the other one has much lower three-fold degenerate state in energy, namely the Tao-Thouless-like state, as increasing the strength of Rashba spin-orbit coupling α_R. It should be point out that the nine-fold degenerate fractional quantum spin Hall state evolved into the three-fold degenerate Tao-Thouless-like state does not undergo a quantum phase transition, since the Tao-Thouless like-state can be adiabatically connected to the fractional quantum spin Hall state by changing the aspect ratio of geometric structure (see the main text). When the Rashba spin-orbit coupling α_R is further increased ($\alpha_R \leq 0.08$), the Tao-Thouless-like state is robust against the interaction between spins. If the Rashba spin-orbit coupling α_R is increased beyond the 0.08, the lowest three-fold degenerate state will be destroyed and evolved into the conventional Fermi

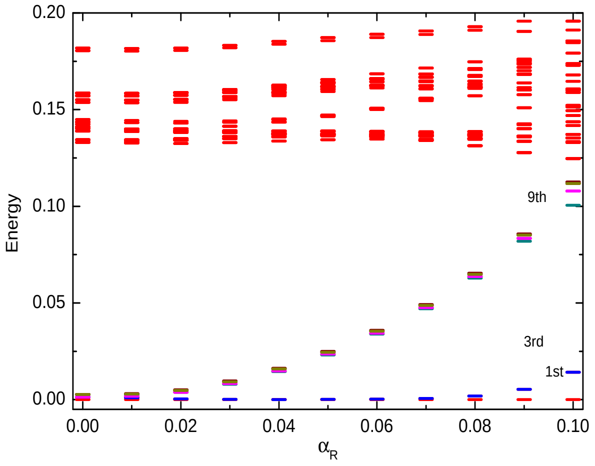

FIG. 3: (Color online) The sixty lowest eigenenergies as a function of interaction α_R for the system size $N_s = 2 \times N_x(= 2) \times N_y(= 6)$. Additionally, these ground state energies are all shifted by E_1, which is the lowest energy for the system.

liquid state.

---

[1] Sheng, D.N., Gu, Z.-C., Sun, K. & Sheng, L. Fractional quantum Hall effect in the absence of Landau levels. *Nat. Commun.* **2**, 389 (2011).

[2] Repellin, C., Bernevig, B.A. & Regnault, N. Z_2 fractional topological insulators in two dimensions. *Phys. Rev. B* **90**, 245401 (2014).

[3] Bernevig, B.A. & Regnault, N. Thin-Torus Limit of Fractional Topological Insulators. *arXiv*:1204.5682 (2012).

[4] Budich, J.C. & Ardonne, E. Fractional topological phase in one-dimensional flat bands with nontrivial topology. *Phys. Rev. B* **88**, 035139 (2013).

[5] Sheng, D.N., Weng, Z.Y., Sheng, L. & Haldane, F.D.M. Quantum Spin-Hall Effect and Topologically Invariant Chern Numbers. *Phys. Rev. Lett.* **97**, 036808 (2006).

[6] Niu, Q., Thouless, D.J. & Wu, Y.S. Quantized Hall conductance as a topological invariant. *Phys. Rev. B* **31**, 3372 (1985).

[7] Laughlin, R.B. Quantized Hall conductivity in two dimensions. *Phys. Rev. B* **23**, 5632 (1981).

[8] Halperin, B.I. Quantized Hall conductance, current-carrying edge states, and the existence of extended states in a two-dimensional disordered potential. *Phys. Rev. B* **25**, 2185 (1982).

[9] Li, W., Sheng, D.N., Ting, C.S. & Chen, Y. Fractional quantum spin Hall effect in flat-band checkerboard lattice model. *Phys. Rev. B* **90**, 081102(R) (2014).

[10] Thouless, D.J., Kohmoto, M., Nightingale, M.P. & Nijs, M. den. Quantized Hall Conductance in a Two-Dimensional Periodic Potential. *Phys. Rev. Lett.* **49**, 405 (1982).
